# Shadow pitching deviates ball release position: kinematic analysis in high school baseball pitchers

**DOI:** 10.1186/s13102-021-00255-7

**Published:** 2021-03-17

**Authors:** Shigeaki Miyazaki, Go Yamako, Koji Totoribe, Tomohisa Sekimoto, Yuko Kadowaki, Kurumi Tsuruta, Etsuo Chosa

**Affiliations:** 1 Rehabilitation Unit, University of Miyazaki Hospital, 5200 Kihara Kiyotake, Miyazaki, Miyazaki 889-1692 Japan; 2 Department of Mechanical Design Systems Engineering, Faculty of Engineering, University of Miyazaki, 1-1 Gakuen Kibanadai-nishi, Miyazaki, Miyazaki 889-2192 Japan; 3 Department of Orthopaedic Surgery, Faculty of Medicine, University of Miyazaki, 5200 Kihara Kiyotake, Miyazaki, Miyazaki 889-1692 Japan; 4 Clinical Research Support Center, University of Miyazaki Hospital, 5200 Kihara Kiyotake, Miyazaki, Miyazaki 889-1692 Japan; 5 School of Nursing, Faculty of Medicine, University of Miyazaki, 5200 Kihara Kiyotake, Miyazaki, Miyazaki 889-1692 Japan

**Keywords:** Shadow pitching, Throwing motion, Kinematic differences, Ball release target, Pitching disorders

## Abstract

**Background:**

Although shadow pitching, commonly called “towel drill,” is recommended to improve the throwing motion for the rehabilitation of pitching disorders before the initiation of a throwing program aimed at returning to throwing using a ball, the motion differs from that of normal throwing. Learning improper motion during ball release (BR) may increase shoulder joint forces. Abnormal throwing biomechanics leads to injures. However, there has been no study of shadow pitching focusing on the BR position. The purpose of the present study was to evaluate the BR position and kinematic differences between shadow pitching and normal throwing. In addition, the effect of setting a target guide for BR position on throwing motion was examined in shadow pitching.

**Methods:**

The participants included in this study were 20 healthy male students who were overhand right-handed pitchers with no pain induced by a throwing motion. Participants performed normal throwing (task 1), shadow pitching using a hand towel (task 2), and shadow pitching by setting a target of the BR position (task 3). A motion capture system was used to evaluate kinematic differences in throwing motions, respectively. Examination items comprised joint angles and the differences in BR position.

**Results:**

BR position of task 2 shifted significantly toward the anterior, leftward, and downward directions compared with task 1. The distance of BR position between tasks 1 and 2 was 24 ± 10%. However, task 3 had decreased BR deviation compared with task 2 (the distance between 3 and 1 was 14 ± 7%). Kinematic differences were observed among groups at BR. For shoulder joint, task 2 showed the highest value in abduction and horizontal adduction among groups. In spine flexion, left rotation and thorax flexion, task 2 was significantly higher than task 1. Task 3 showed small differences compared with task 1.

**Conclusions:**

The BR position of shadow pitching deviated significantly in the anterior, leftward, and downward directions compared with normal throwing. Furthermore, we demonstrated that the setting of BR target reduces this deviation. Thus, the target of BR position should be set accurately during shadow pitching exercises in the process of rehabilitation.

## Background

The throwing motion in baseball is a full-body exercise. It requires a kinetic chain among each joint of the lower limbs, the trunk, and the upper limbs. Kinetic energy is transmitted into a ball through the chain [1–4]. Pitching disorders caused by abnormal throwing biomechanics can be induced by the dysfunction of even a single joint. In addition, Chalmers et al. [5]. reported that altered knee flexion at ball release, early trunk rotation, loss of shoulder rotational range of motion, and increased elbow flexion at ball release may increase shoulder and elbow torques and risk for injury. Therefore, abnormal throwing biomechanics leads to injuries in the shoulder and elbow joints [6, 7].

Biomechanical studies on throwing motion have evaluated joint angles, forces, and torques using an optical motion capture system and have indicated correlations to injuries [6, 8–12]. Excessive shoulder horizontal abduction [13] increases the anterior joint force during maximum shoulder external rotation (MER) [11], resulting in internal impingement, rotator cuff tears, and superior labral anterior to posterior lesions [14]. Maximum shoulder joint force is reached around the time of ball release (BR) [15, 16]. Fleisig et al. [15] reported that 1090 N of shoulder compressive force was produced after BR. The risk of glenoid labrum injury may increase with level of competition, labrum injury may also occur from the combination of humeral translation, compression, and internal rotation [15]. These studies indicate the importance of establishing a proper throwing motion to prevent injuries and encourage the investigation of throwing kinematics during rehabilitation and training.

Shadow pitching, commonly called “towel drill,” is recommended to improve the throwing motion for the rehabilitation of pitching disorders before the initiation of a throwing program aimed at returning to throwing using a ball [17, 18]. Shadow pitching is also performed as a warm-up exercise before throwing of the ball. The pitchers practice the throwing motion by holding a hand towel with their fingers. However, in daily clinical practice, there seems to be a difference in the motion between shadow pitching and normal throwing using a ball. Specifically, the BR position of shadow pitching may deviate to the anterior directions compared with normal throwing because there is no BR target. A previous biomechanical study showed that the glenohumeral external rotation angle was significantly lower in shadow pitching than in normal throwing at MER [19]. However, there has been no study of shadow pitching focusing on the BR position. Changes in BR position may induce greater shoulder joint forces [12].

The purpose of the present study was to evaluate the BR position and kinematic differences between shadow pitching and normal throwing using an optical motion capture system. In addition, the effect of setting a target guide for BR position on throwing motion was examined in shadow pitching. We hypothesized that the BR position of shadow pitching deviates to the anterior directions compared to normal throwing using a ball. We also hypothesized that the target of BR position reduces the kinematic differences between normal and shadow pitching.

## Methods

### Design

This study used a controlled laboratory design with counterbalanced conditions in a laboratory setting. Primary independent variable was task (normal throwing, shadow pitching using a hand towel, and shadow pitching by setting a target of the BR position). Primary outcomes included various joints angles and the BR position.

### Participants

The participants included in this study were 20 healthy male students (age, 16.5 ± 0.8 years (mean ± standard deviation [SD]); height, 1.72 ± 0.05 m; weight, 64.8 ± 7.2 kg) who were right-handed pitchers with no pain induced by a throwing motion. No participants were injured or recovering from an injury at the time of testing, and none had previous shoulder or elbow surgery. This study was conducted after receiving the approval of the Research Ethics Committee of the Faculty of Medicine, University of Miyazaki. This study was conducted in accordance with the Declaration of Helsinki. Because the subjects were minors, we explained the purpose and the content of the study orally and in written form to the subjects and their parents, and obtained written informed consent.

### Procedures

**Fig. 1:**
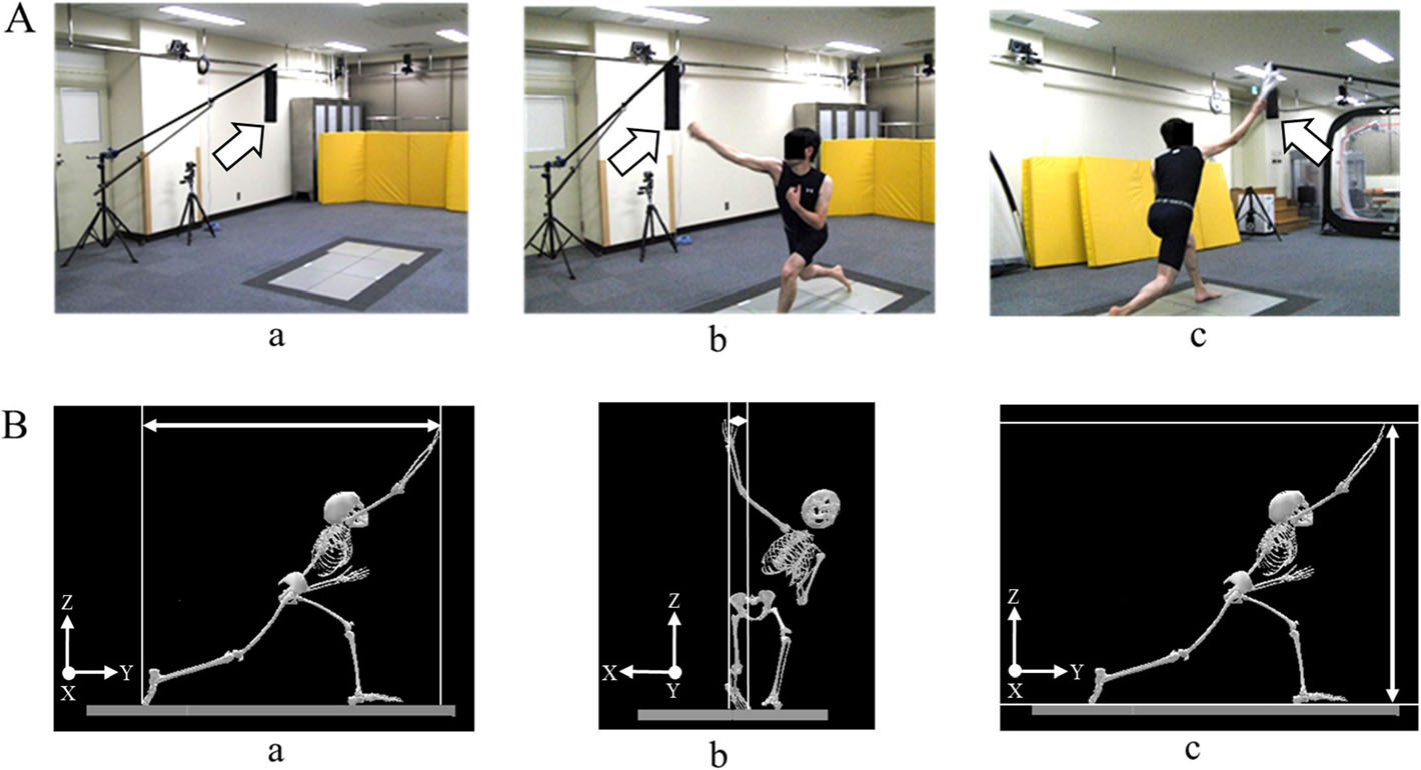
(**a**) Setting a target of ball release (BR) position (arrows). The BR target was placed with reference to the BR position of normal throwing using a ball. a: Setting the target of BR position, b: Left-side view of shadow pitching, c: Posterior view of shadow pitching. (**b**) Definition of BR position. BR position was defined as a distance to the right finger from the right toe at BR position. a: Direction of anterior/posterior, b: Transverse (left/right) direction, c: Vertical (upward/downward) direction

The participants performed three tasks of throwing motions in the following order. Task 1 performed normal throwing using a ball (approximately 146 g), task 2 performed shadow pitching using a hand towel (66 g, 30 cm in length), and task 3 performed shadow pitching using a hand towel with a BR position setting. In task 3, a target of BR position was set by placing an artificial leather sheet (40 cm in length, 10 cm in width) (Fig. 1A). Fastball was the method of gripping wherein the first to fourth fingers were placed on the seam of the ball and the first finger was placed at the center of the ball. The towel was rounded to hold. As far as possible, the grip on the towel was similar to that on the ball.

Throwing motions were measured using an optical motion capture system (Vicon Nexus, Vicon Motion Systems, London, UK) with 10 infrared cameras (MX 3 and MXT 20, Vicon Motion Systems). The subjects were no clothes on their upper body but wore skin-tight clothes (Under Armour, Maryland, USA) on their lower body. Thirty-five reflective markers (14-mm diameter) were attached to the participants according to the Plug-in-Gait model protocol [20, 21]. The sampling frequency of the system was set at 250 Hz. Two video cameras (Bonita Video Camera Bonita 720c, Vicon Motion Systems) were also used with a sampling frequency of Procedures125 Hz, synchronized with the motion capture system. A global coordinate system was established, denoting the landmarks of a baseball field. The Y-axis is oriented from the mound toward the catcher and the X-axis from the first base toward the third base, which is orthogonal to the Y-axis. Further, the Z-axis is vertically oriented upward (Fig. 1B).

The measurement of throwing motions was performed indoor using a practice net (2ZM595, Mizuno, Osaka, Japan) placed six meters in front of the support leg at wind up. The subjects warmed up and practiced so that they would pitch naturally. Before testing, the subjects completed a preparation routine, including stretching, and warm-up throwing. They were asked to pitch the ball as fast as possible. Instructions to quantify the throwing motion included 1) throwing in a pitching motion, 2) no wind up throwing (a throwing technique in which the pitcher throws the ball by stopping at the thoracic level or hip), 3) fastball as the pitch type, and 4) a 20-s interval between pitches. Each throwing task was performed five times in each participant.

### Data processing

Using the plug-in gait model, kinematic data were obtained from positions of each reflective marker [20, 21]. The throwing kinematics were evaluated at MER and BR, and BR time was determined using a video camera. The BR time for shadow pitching was defined as the time when the extension of the elbow reached the maximum value. The elbow extension was the highest during the BR phase while throwing the ball [22]. Kinematic examination items comprised the joint angles of the shoulder (horizontal adduction/horizontal abduction, abduction/adduction, and internal rotation/external rotation), elbow (flexion/extension), hip (flexion/extension, adduction/abduction, and internal rotation/external rotation), knee (flexion/extension), ankle (dorsiflexion/plantar flexion), spine (flexion/extension, lateral bending, and rotation), thorax (flexion/extension, left/right lateral bending, and left/right rotation), and pelvis (anterior/posterior tilt, left/right turn, and left/right rotation).

The differences in BR position among the tasks were assessed. The BR position was BR position was defined as the distance from the second metatarsal head of the right foot to the second metacarpal head of the right hand in anterior/posterior, transverse (left/right), and vertical (upward/downward) directions (Fig. 1B) based on the global coordinate system. BR position was normalized by the subject’s body height. The stride length of throwing was not significantly different among the tasks.

The throwing type of participants was evaluated using the arm slot angle based on the study by Escamilla et al. [23]. The angle was divided into three throwing groups: overhand (≤40° arm slot angle), three-quarter (41° to 69° arm slot angle), and sidearm (≥70° arm slot angle).

### Statistical analysis

A statistical power analysis was performed using EZR software version 1.38 (Saitama Medical Center, Jichi Medical University, Saitama, Japan) [24]. We conducted a sample size calculation based on the power of the corresponding t-test with a primary endpoint of the right shoulder horizontal adduction angle, which greatly affects the deviation of the BR position. The SD of the right shoulder horizontal adduction angle at BR position in tasks 1 and 2 was nine degrees, and the value for task 2 was expected to be at least seven degrees greater than that of task 1. We conducted a sample size calculation (α = 0.05, power = 0.8) based on SD = 9 and Δ = 7 (difference in the means for both tasks) as estimated. The results indicated that the required sample size was 16. To allow for some withdrawals, we set the required number of subjects at 20.

In order to analyze the motion of the best performance, data from the motion, which the subjects were most satisfied with, were used as representative values. All data were presented as mean ± SD. Analysis software, IBM SPSS Version 21 (IBM Corp., Armonk, NY, USA) was used. After confirming the data followed a normal distribution, we performed one- factor repeated measures analysis of variance (ANOVA). For the items in which a significant difference was observed, Bonferroni correction of the corresponding t-test was applied for comparison. The significance level was set at 0.05.

## Results

Seventeen participants were classified as the three-quarter (mean angle of the arm slot angle: 59.3 ± 7.7°) and three were sidearm (75.5 ± 4.7°).

Significant differences were noted in BR position among tasks (Table 1). The BR position of task 2 significantly deviated toward the anterior, leftward, and downward directions compared with task 1. The distance of BR position between tasks 1 and 2 was 24 ± 10%. However, task 3 had decreased BR deviation compared with task 2 (the distance between 3 and 1 was 14 ± 7%).

**Table 1:** The difference in the BR position among tasks

	Arm Acceleration				
	BR				
Direction of BR position	Task 1	Task 2	Task 3	One factor repeated measures ANOVA	Bonferroni correction *p*-value
Anterior (+) or Posterior (−)	78 ± 8	91 ± 6 ^a^	86 ± 9 ^b c^	F = 66.206, *p* < .001	< .001 ^a c^, .001 ^b^
Transverse: Right (+) or Left (−)	14 ± 11	4 ± 8 ^a^	7 ± 8 ^b c^	F = 25.836, *p* < .001	< .001 ^a^, .015 ^b^, .001 ^c^
Vertical: Upward (+) or Downward (−)	76 ± 7	59 ± 10 ^a^	70 ± 8 ^b c^	F = 42.588, *p* < .001	< .001 ^a b^, .001 ^c^

All values are in percentages. Values are presented as mean ± standard deviation

*Abbreviation*: *BR* Ball release, *ANOVA* Analysis of variance

^a^ Significant difference between task 1 and task 2

^b^ Significant difference between task 2 and task 3

^c^ Significant difference between task 1 and task 3

**Fig. 2:**
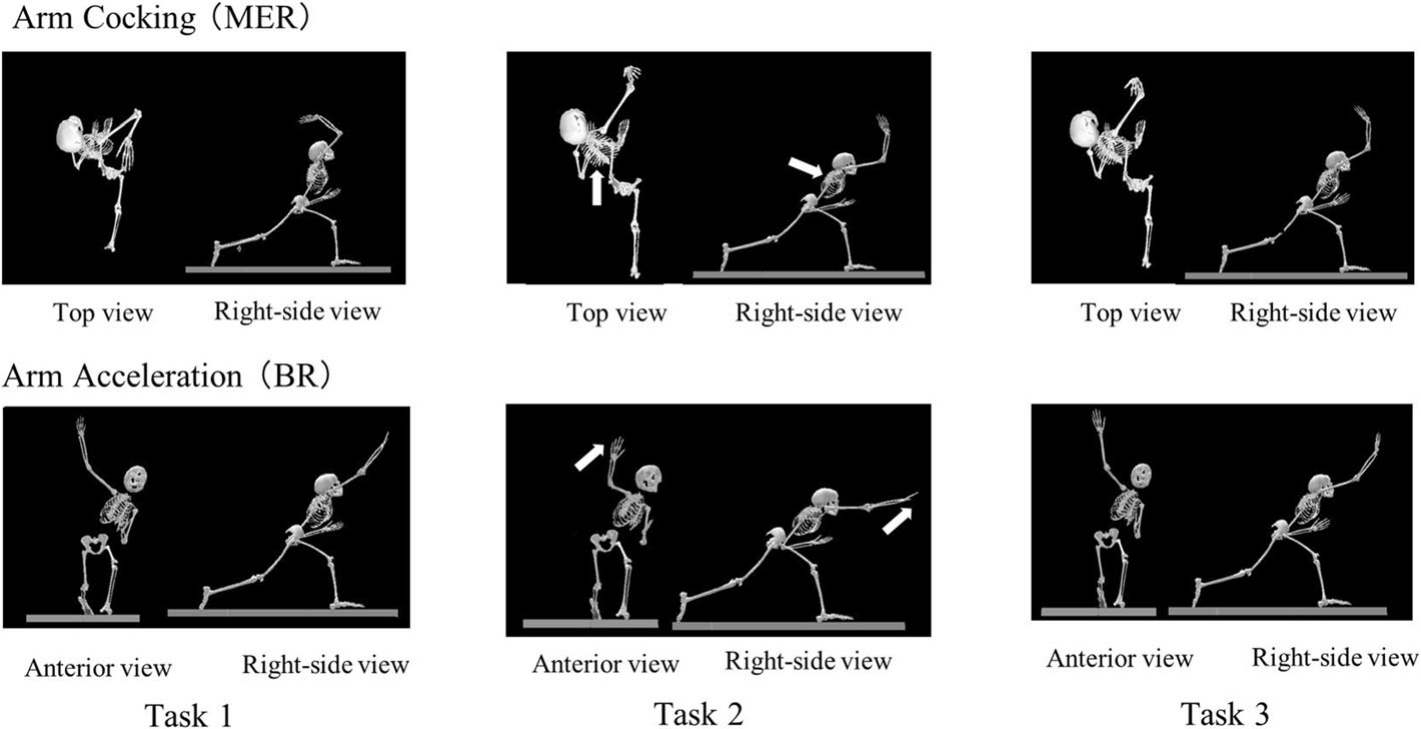
The arm cocking (MER) and arm acceleration (BR) positions for each task. At the MER, the throwing motion of task 2 was not accurately performed in the spinal column and thoracic extension position compared with tasks 1 and 3 (arrows). At the BR position, the leaning of upper limbs of task 2 was greater than those of tasks 1 and 3. Abbreviation: *MER* maximum shoulder external rotation, *BR* ball release

Significant differences were observed among tasks in various joints angles at BR (Table 2). In the shoulder joint of the throwing side, task 2 showed the highest value in abduction and horizontal adduction and the lowest value in external rotation. In spine flexion, left rotation, and thorax flexion, task 2 was significantly higher than task 1. Task 3 showed small differences compared with tasks 1 (Table 2 and Fig. 2).

**Table 2:** Joint angles at BR position

	Arm Acceleration				
	BR				
Direction of Motion	Task 1	Task 2	Task 3	One-factor repeated measures ANOVA	Bonferroni correction *p*-value
Right Shoulder					
Horizontal Adduction (+) or Abduction (−)	2.3 ± 7.9	17.3 ± 9.2 ^a^	7.8 ± 7.9 ^b c^	F = 36.902, *p* < .001	< .001 ^a b^, .001 ^c^
Abduction (+) or Adduction (−)	98.1 ± 7.2	103.2 ± 6.2 ^a^	100.1 ± 9.1	F = 5.036, *p* = .023	< .001 ^a^
Internal Rotation (+) or External Rotation (−)	− 91.8 ± 16.3	− 78.8 ± 21.4 ^a^	− 85.3 ± 15.7 ^c^	F = 11.496, *p* < .001	.002 ^a^, .041 ^c^
Spine					
Flexion (+) or Extension (−)	11.4 ± 7.4	23.3 ± 7.9 ^a^	18.9 ± 8.0 ^c^	F = 26.104, *p* < .001	< .001 ^a^, .002 ^c^
Lateral Bending: Left (+) or Right (−)	32.7 ± 9.8	28.7 ± 9.0 ^a^	30.0 ± 7.7	F = 6.078, *p* = .005	.007 ^a^
Rotation: Left (+) or Right (−)	17.7 ± 7.3	23.2 ± 6.9 ^a^	21.1 ± 8.1	F = 7.437, *p* = .002	.007 ^a^
Thorax					
Flexion (+) or Extension (−)	32.3 ± 9.8	42.0 ± 8.6 ^a^	37.7 ± 9.1 ^b c^	F = 21.538, *p* < .001	< .001 ^a^, .030 ^b^, .002 ^c^

All angles are in degrees. Values are presented as mean ± standard deviation

*Abbreviation*: *BR* Ball release, *ANOVA* Analysis of variance

^a^ Significant difference between task 1 and task 2

^b^ Significant difference between task 2 and task 3

^c^ Significant difference between task 1 and task 3

Significant differences were observed among tasks in various joint angles at MER (Table 3). In the shoulder joint of the throwing side, task 2 showed the highest horizontal adduction and lowest external rotation. Evaluation of horizontal adduction showed no significant difference between tasks 1 and 3. Thorax and spine flexion and spine left rotation were significantly higher in task 2 than in task 1 (Table 3 and Fig. 2), and spine flexion and thorax flexion of task 3 were significantly lower than those of task 2.

**Table 3:** Joint angles at MER

	Arm Cocking				
	MER				
Direction of Motion	Task 1	Task 2	Task 3	One-factor repeated measures ANOVA	Bonferroni correction *p*-value
Right Shoulder					
Horizontal Adduction (+) or Abduction (−)	3.5 ± 6.0	12.6 ± 9.0 ^a^	5.0 ± 7.9 ^b^	F = 14.768, *p* < .001	.001 ^a b^
Internal Rotation (+) or External Rotation (−)	− 160.0 ± 13.9	− 149.7 ± 14.3 ^a^	− 153.7 ± 12.6 ^b c^	F = 24.664, *p* < .001	< .001 ^a^, .008 ^b^, .002 ^c^
Spine					
Flexion (+) or Extension (−)	−6.6 ± 8.2	6.4 ± 10.2 ^a^	0.0 ± 10.3 ^b c^	F = 24.748, *p* < .001	< .001 ^a^, .003 ^b^, .011 ^c^
Rotation: Left (+) or Right (−)	6.3 ± 9.2	12.0 ± 8.0 ^a^	9.3 ± 8.7	F = 8.436, *p* = .001	.005 ^a^
Thorax					
Flexion (+) or Extension (−)	21.0 ± 8.4	30.8 ± 9.9 ^a^	25.7 ± 9.0 ^b c^	F = 24.572, *p* < .001	< .001 ^a^, .002 ^b^, .006 ^c^

All angles are in degree. Values are presented as mean ± standard deviation

*Abbreviation*: *MER* Maximum shoulder external rotation, *ANOVA* Analysis of variance

^a^ Significant difference between task 1 and task 2

^b^ Significant difference between task 2 and task 3

^c^ Significant difference between task 1 and task 3

## Discussion

During rehabilitation, pitchers need to stabilize their physical status and obtain proper throwing motion to return to pitching. This study evaluated motion differences between shadow pitching and normal throwing. Compared to normal throwing, the BR position deviates in shadow pitching, resulting in different kinematics at BR and MER between shadow pitching and normal throwing. This improper throwing of leaning the upper body forward increased spine rotation and shoulder horizontal adduction during the acceleration phase. However, the kinematics of shadow pitching with the target setting of BR position was similar to that of normal throwing.

In this study, the shoulder horizontal adduction angle of normal throwing at BR was within the range of values in reported in previous studies [15, 25–28]. Fleisig et al. [26] reported that 33 high school pitchers showed 10° shoulder horizontal adduction at BR. Avoiding excessive shoulder angles should be emphasized. Tanaka et al. [12] reported that the minimum anterior–posterior and vertical shear forces on the shoulder joint were at 10.7° for shoulder horizontal adduction and 80.6° for shoulder abduction at BR. A 5° deviation from these shoulder angles indicates significantly increased joint forces. Our study demonstrated that shadow pitching showed excessive horizontal adduction and abduction compared with normal throwing (15° difference in horizontal adduction, 5° difference in abduction). However, the target of BR position (5.5° deviation in horizontal adduction, 2° deviation in abduction, compared with normal throwing) decreases excessive shoulder motions. Therefore, the target of BR position is required in shadow pitching during rehabilitation.

The MER angle of shadow pitching was significantly lower than that of normal throwing. This finding is consistent with a previous study by Okamoto et al. [19]. The shoulder external rotation during the throwing motion is explained as a lagging back phenomenon in which the movement of the forearm and hand segment lag behind the upper segment when switching from arm cocking to arm acceleration [3, 10]. There may be reduced MER angle with shadow pitching because of the reduced moment due to the lack of the ball mass. In addition, this mass helps keep the upright posture of the upper body during the acceleration phase. The extension in the thorax and spine at MER was significantly lower in shadow pitching than in normal throwing. This indicates that shadow pitching was not able to stretch their chest during the acceleration phase, which may increase the shoulder joint loads. Not only glenohumeral external rotation but also scapular posterior tilt and thoracic extension contribute to the shoulder external rotation during the throwing motion [10]. These restrictions of movement have a greater effect on loads acting on the glenohumeral joint and can lead to pitching disorders.

From our results on shadow pitching biomechanics, the following points are crucial to create an effective rehabilitation program for pitching disorders: (1) recognizing the gap of kinematics between shadow pitching and normal throwing using a ball; (2) ensuring sufficient flexibility of the spine and the thorax; (3) setting a target of BR position during shadow pitching exercises; (4) transitioning to throwing using a ball after learning the appropriate BR position in shadow pitching exercise. Being aware of these points will reduce the differences between shadow pitching and normal throwing using and can facilitate safer and more effective rehabilitation.

The study has some limitations. First, data collection was done in a laboratory setting. Our laboratory did not have a regulation mound and there was not enough space for the throwing distance. Moreover, the subjects performed throwing without shoes. Therefore, throwing performance may have been affected by these conditions [29, 30]. Second, the age of the participants is about 16.5 years old. Their throwing motion is not mature, which may cause variability of the kinematics [31]. Third, skin movement artifact for positions of reflective markers did not completely disappear [32, 33]. This may cause errors in calculating kinematic parameters. Fourth, a sampling rate of 250 Hz may not have been enough to calculate kinetic parameters around MER and BR. Our further studies will focus on kinetics of shadow pitching.

## Conclusions

In summary, our kinematic analysis demonstrated that the BR position of shadow pitching deviated significantly to the anterior, leftward, and downward directions compared with normal throwing. Furthermore, we showed that the setting of target of BR position can reduce this deviation. Thus, the target of BR position should be set accurately during shadow pitching exercises in the process of rehabilitation.

## Data Availability

The datasets used and/or analyzed during the current study are available from the corresponding author on reasonable request.

## Abbreviations

ANOVA: Analysis of variance
BR: Ball release
MER: Maximum shoulder external rotation
SD: Standard deviation
